# Prior cervical cytology and high-risk HPV testing results for 311 patients with invasive cervical adenocarcinoma: a multicenter retrospective study from China’s largest independent operator of pathology laboratories

**DOI:** 10.1186/s12879-019-4614-y

**Published:** 2019-11-11

**Authors:** Fengxiang Xie, Liran Zhang, Dongman Zhao, Xuefen Wu, Mingsong Wei, Xuelian Zhang, Xiaohui Wu, Hao Fang, Xue Xu, Meng Yang, Debo Qi

**Affiliations:** 1 Department of Pathology, Jinan KingMed Diagnostics, Jinan, 250101 Shandong Province China; 2 Department of Pathology, Kunming KingMed Diagnostics, Kunming, 650000 Yunnan Province China; 3 Department of Pathology, Guangxi KingMed Diagnostics, Nanning, 530007 Guangxi Autonomous Region China; 4 Department of Pathology, Nanjing KingMed Diagnostics, Nanjing, 250032 Jiangsu Province China; 5 Department of Pathology, Chongqing KingMed Diagnostics, Chongqing, 400050 Chongqing Municipality China; 6 Department of Pathology, Changsha KingMed Diagnostics, Changsha, 410205 Hunan Province China; 7 Department of Pathology, Hefei KingMed Diagnostics, Hefei, 230088 Anhui Province China; 8 Department of Pathology, Zhengzhou KingMed Diagnostics, Zhengzhou, 450000 Henan Province China; 9 Department of Genetics, Jinan KingMed Diagnostics, Jinan, 250101 Shandong Province China

**Keywords:** Cervical adenocarcinoma, Cytology, High-risk human papillomavirus (HR-HPV) testing, Co-testing, Cervical cancer screening

## Abstract

**Background:**

High-risk human papillomavirus (HR-HPV) testing is more sensitive than cytology for the detection of cervical cancer and its precursors. However, limited and inconsistent data are available about the efficacy of the combination of these two methods for screening cervical adenocarcinoma. This multicenter retrospective study investigated the screening results of a cohort of Chinese patients who were subsequently diagnosed with invasive cervical adenocarcinoma, with the goal of identifying the optimal cervical adenocarcinoma screening method.

**Methods:**

We retrospectively retrieved and analyzed the data from patients with histologically confirmed primary invasive cervical adenocarcinoma from eight local pathology laboratories operated by KingMed Diagnostics, the largest independent operator of pathology laboratories in China, over a 2-year period. Only patients who underwent cytology and/or HR-HPV testing within 6 months before the adenocarcinoma diagnosis were included. HR-HPV DNA was detected using one of two HPV test kits: the Hybrid Capture 2 (HC2) assay (Qiagen, Hilden, Germany) and an HPV genotyping panel (Yaneng Bio, Shenzhen, China).

**Results:**

Of the 311 patients, 136 underwent cytology alone, 106 underwent HR-HPV testing alone, and 69 underwent cytology and HR-HPV co-testing. The sensitivities of cytology alone (64.0, 95% confidence interval [CI]: 55.9–72.0) and HR-HPV testing alone (66.0, 95% CI: 57.0–75.1) were similar (*P* = 0.738). The sensitivity of cytology and HR-HPV co-testing (87.0, 95% CI: 79.0–94.9) was significantly higher than that of either cytology (*P* = 0.001) or HR-HPV testing alone (*P* = 0.002).

**Conclusions:**

Both cytology alone and HR-HPV testing alone showed poor screening efficiency, whereas the combination of the two clearly increased the efficiency of primary cervical adenocarcinoma screening. Thus, cytology and HR-HPV co-testing might be the most efficient cervical adenocarcinoma screening method.

## Background

Cervical cytology has successfully lowered the incidence and mortality rates of cervical cancer in nations where organized cytological screening programs have been well implemented [1–5]. However, the effectiveness of cytological screening is predominantly attributable to a reduction in the incidence of cervical squamous cell carcinoma rather than adenocarcinoma [1–5]. Adenocarcinoma is much less common than squamous cell carcinoma, accounting for only approximately 7–17% of all cervical malignancies [6–8]. In nations with well-organized screening programs, the proportion of cervical adenocarcinoma has risen in recent decades [1–5]. This trend is attributable not only to a marked decrease in the incidence of cervical squamous cell carcinoma but also to an only slightly decreasing, stable, or even increasing absolute incidence of cervical adenocarcinoma [1–5]. These trends in the incidence of cervical squamous cell carcinoma and adenocarcinoma suggest that the incidence of cervical adenocarcinoma is less influenced by cytological screening than that of squamous cell carcinoma, and thus, a screening strategy based solely on cytology might be insufficient to prevent cervical adenocarcinoma.

High-risk human papillomavirus (HR-HPV) infection has been identified as the main cause of cervical cancer, especially, squamous cell carcinoma and its precancerous lesions in women [9, 10]. Several large, long-term, randomized clinical trials [11–15] have confirmed that when used for primary screening, HR-HPV testing is more effective than cervical cytology for the detection of overall cervical carcinoma and its precursors. In addition, the molecular detection of HPV is more objective and easier to automate, which can greatly improve the cancer-screening efficacy [16]. Based on this, HR-HPV detection, either alone or in conjunction with cytology, has been approved as a primary cervical screening modality in many developed nations [17, 18]. However, HPV infection is less prevalent in adenocarcinoma than in squamous cell carcinoma, with the former showing HPV-infection rates ranging from 60.0 to 85.8% [6, 19–23]. Accordingly, it is reasonable to postulate that the new primary screening strategy based on HR-HPV detection alone may be ineffective for the detection of cervical adenocarcinoma.

Globally, data on current screening practices for invasive cervical adenocarcinoma are very limited. Due to the extreme rarity of invasive adenocarcinoma, none of the aforementioned clinical studies [11–15] have been sufficiently powered to determine which method—cytology or HPV detection—is more effective for cervical adenocarcinoma screening. Given that the screening efficiencies of both cytology alone and HR-HPV testing alone are poor, we speculate that the combination of the two tests may increase the effectiveness of primary cervical adenocarcinoma screening. To date, however, only several small retrospective studies have explored the roles of cytology, HR-HPV testing, and their combination for cervical adenocarcinoma screening, and the results have been inconsistent [7, 24–26]. Therefore, we designed the present multicenter retrospective study to evaluate the previous cytology and HR-HPV testing results in a population of Chinese patients with a histological diagnosis of invasive cervical adenocarcinoma. Over a 2-year period, we documented the cytology and HR-HPV testing results obtained within 6 months of the adenocarcinoma diagnosis from eight local pathology laboratories operated by KingMed Diagnostics. HR-HPV DNA was detected using one of two HPV test kits: the Hybrid Capture 2 (HC2) assay (Qiagen, Hilden, Germany) and an HPV genotyping panel (Yaneng Bio, Shenzhen, China). The goal of this investigation was to determine the advantages and limitations of cytology, HR-HPV testing, and cytology and HR-HPV co-testing in detecting invasive cervical adenocarcinoma, and to provide further evidence for formulating a better prevention strategy for cervical adenocarcinoma.

## Patients and methods

### Patient enrollment

KingMed Diagnostics is the largest independent operator of pathology laboratories in China, with local pathology laboratories in every province and municipality. After obtaining approval from the institutional academic board of KingMed Diagnostics, we searched the databases of eight local pathology laboratories of the authors for the records of all patients who had received a histological diagnosis of invasive cervical adenocarcinoma between January 2017 and December 2018. The academic board waived the need for patient consent, as this was an anonymous analysis of retrospective data. Considering the rapid progression to invasive adenocarcinoma reported in a cohort of patients with an interval of less than 3 years between screening and diagnosis [27, 28], we included only patients who had undergone cytological and/or HR-HPV testing within 6 months before the primary diagnosis of cervical adenocarcinoma [29]. All cytology, HPV testing, and histological examinations had been performed at the local pathology laboratories of KingMed Diagnostics.

### Cytology preparation and interpretation

Cytology tests were performed using liquid-based cytology methods, and all procedures were strictly in accordance with the manufacturers’ instructions, as we have previously reported [7, 30]. The cytology results were classified as follows: negative for intraepithelial lesion or malignancy (NILM), atypical squamous cells of undetermined significance (ASC-US), low- or high-grade squamous intraepithelial lesion (LSIL and HSIL, respectively), atypical squamous cells—cannot exclude HSIL (ASC-H), atypical glandular cells (AGC), and cervical cancer cells.

### HPV testing

HPV DNA was detected using one of two HPV test kits: the HC2 assay (Qiagen, Hilden, Germany) and an HPV genotyping panel (Yaneng Bio, Shenzhen, China) [7, 31]. The HC2 HPV assay uses nucleic acid hybridization and signal amplification with chemiluminescence, and can semi-quantitatively detect 13 HR-HPV genotypes (16, 18, 31, 33, 35, 39, 45, 51, 52, 56, 58, 59, and 68). The HPV genotyping panel [7, 31] is an in vitro diagnostic assay that uses the polymerase chain reaction (PCR)-reverse dot blot hybridization method. This multiplex PCR technique can concurrently detect 23 HPV subtypes (14 HR-HPV genotypes: 16, 18, 31, 33, 35, 39, 45, 51, 52, 56, 58, 59, 66, and 68; 9 low- or undetermined-risk HPV genotypes: 6, 11, 42, 43, 53, 73, 81, 82, and 83).

### Statistical analysis

All statistical analyses were performed using SPSS software (version 19.0, IBM Co., Chicago, IL, USA). Categorical data were compared using the Pearson χ^2^ test, while continuous data were compared using the Student *t*-test or one-way analysis of variance, where appropriate. *P* < 0.05 was considered statistically significant.

## Results

### Patient cohort

A total of 311 patients had undergone cervical cancer screening within 6 months before the adenocarcinoma diagnosis. The number of patients in each laboratory ranged from 19 to 88 (mean, 34.5; median, 33.5). In total, 136 (43.7%) patients (average age, 49.3 years; range, 27–80 years) had undergone cytology alone, 106 (34.1%) patients (average age, 47.0 years; range, 27–82 years) had undergone HR-HPV testing alone, and 69 (22.2%) patients (average age, 49.3 years; range, 31–78 years) had undergone combined cytology and HR-HPV testing. No obvious differences were observed in the average ages of the patients who underwent these three screening modalities (*P* = 0.143).

### Results of cytology alone

Among the 136 women who underwent cytology alone, the average interval between cytology and the primary adenocarcinoma diagnosis was 37.8 days (range, 0–180 days). In 88.2% (120 patients) of these women, cytology had been performed within 1 month before the histological diagnosis. Overall, abnormal findings were present in 64.0% (87/136) of the patients (Table 1). In 26.5% (36/136) patients, the cytological findings were interpreted as AGC, which was the most common abnormal cytology result. A cytology report of carcinoma was rendered in another 8.1% (11/136) patients. In the remaining 29.4% (40/136) patients, other abnormal results were reported such as ASC-US, LSIL, ASC-H, and HSIL.

**Table 1:** Results of cytology alone performed within 6 months before diagnosis of invasive cervical adenocarcinoma

Cytology interpretation	No.	%
NILM	45	33.1
Unsatisfactory	4	2.9
ASC-US	11	8.1
ASC-H	12	8.8
LSIL	1	0.7
HSIL	16	11.8
Cancer cells	11	8.1
AGC	36	26.5
Total	136	100.0

*NILM*, Negative for intraepithelial lesion or malignancy; *ASC-US*, Atypical squamous cells of undetermined significance; *ASC-H*, Atypical squamous cells—cannot exclude *HSIL; LSIL*, Low-grade squamous intraepithelial lesion; *HSIL*, High-grade squamous intraepithelial lesion; *AGC*, Atypical glandular cells

### Results of HPV testing alone

Of the 106 patients who underwent HPV testing alone, 57 (53.8%) underwent HC2 HR-HPV testing and 49 (46.2%) underwent genotyping detection (Table 2). In 86.0% (49/57) of the patients who underwent HC2 testing and 89.8% (44/49) of the patients who underwent genotyping detection, the HPV test was performed within 1 month before cancer diagnosis. The average interval between HR-HPV testing and primary adenocarcinoma diagnosis did not significantly differ (*P* = 0.136) between the patients who underwent HC2 testing (14.2 days; range, 0–95 days) and those who underwent genotyping detection (8.0 days; range, 0–69 days). Overall, HR-HPV infection was detected in 66.0% of the patients who underwent HPV testing alone (Table 3). The positive rates of HR-HPV were similar for the two HPV test methods (HC2 testing: 63.2% [36/57] and genotyping detection: 69.4% [34/49]; *P* = 0.500).

**Table 2:** Results of HPV testing performed within 6 months before diagnosis of invasive cervical adenocarcinoma

HPV testing	Positive		Negative	
	No.	%	No.	%
HC2 assay (*n* = 57)	36	63.2	21	36.8
Genotyping (*n* = 49)	34	69.4	15	30.6
Total (*n* = 106)	70	66.0	36	34.0

*HR-HPV*, High-risk human papillomavirus; *HC2*, Hybrid Capture 2

**Table 3:** Results of cytology and HR-HPV co-testing performed within 6 months before histological diagnosis of invasive cervical adenocarcinoma

Cytology	HC2 testing (*n* = 24)		Genotyping (*n* = 45)		Total (*n* = 69)	
	Negative (%)	Positive (%)	Negative (%)	Positive (%)	Negative (%)	Positive (%)
NILM	3 (12.5)	6 (25.0)	6 (13.3)	1 (2.2)	9 (13.0)	7 (10.1)
Unsatisfactory	0 (0)	1 (4.2)	0 (0)	1 (2.2)	0 (0)	2 (2.9)
ASC-US	1 (4.2)	4 (16.7)	3 (6.7)	1 (2.2)	4 (5.8)	5 (7.2)
ASC-H	0 (0)	2 (8.3)	1 (2.2)	5 (11.1)	1 (1.4)	7 (10.1)
HSIL	0 (0)	0 (0)	0 (0)	2 (4.4)	0 (0)	2 (2.9)
Cancer cells	0 (0)	2 (8.3)	2 (4.4)	8 (17.8)	2 (2.9)	10 (14.5)
AGC	2 (8.3)	3 (12.5)	5 (11.1)	10 (22.2)	7 (10.1)	13 (18.8)
Total	6 (25.0)	18 (75.0)	17 (37.8)	28 (62.2)	23 (33.3)	46 (66.7)

*HR-HPV*, High-risk human papillomavirus; *HC2*, Hybrid Capture 2; *NILM*, negative for intraepithelial lesion or malignancy; *ASC-US*, Atypical squamous cells of undetermined significance; *ASC-H*, Atypical squamous cells—cannot exclude *HSIL; HSIL*, High-grade squamous intraepithelial lesion; *AGC*, Atypical glandular cells

### Results of cytology and HR-HPV co-testing

Of the 69 women who had undergone cytology and HR-HPV co-testing, 67 (97.1%) had undergone cytology and 66 (95.7%) had undergone HPV testing within 1 month before adenocarcinoma diagnosis. The average interval between testing and the primary histological diagnosis did not significantly differ between the two test methods (cytology: 7.9 days; range, 0–96 days and HPV testing: 8.8 days; range, 0–167 days; *P* = 0.773). Of the patients who underwent co-testing, 24 (34.8%) underwent HC2 testing and 45 (65.2%) underwent HPV genotyping detection. The average test–diagnosis interval was slightly but not significantly higher for HC2 testing (16.3 days; range, 0–167 days) than for genotyping detection (4.9 days; range, 0–44 days; *P* = 0.152).

Abnormal findings were detected in 73.9% (51/69) of patients on cytology, 66.7% (46/69) of patients on HPV testing, and 87.0% (60/69) of patients on both cytology and HPV testing (Table 3). Only 37 (53.6%) patients had abnormal findings on concurrent cytology and HPV testing; 9 (13.0%) patients had negative results on both cytology (NILM) and HR-HPV testing; 14 (20.3%) patients had abnormal cytology results but negative HR-HPV testing results; and 7 (10.1%) patients had negative cytology results (NILM) but positive HR-HPV testing results.

### Comparison of different screening modalities

Cytology and HR-HPV co-testing (87.0% [60/69], 95% confidence interval [CI]: 79.0–94.9) was significantly more sensitive than cytology alone (64.0% [87/136], 95% CI: 55.9–72.0; *P* = 0.001) and HR-HPV testing alone (66.0% [70/106], 95% CI: 57.0–75.1; *P* = 0.002). However, the sensitivity of cytology alone was similar to that of HR-HPV testing alone (*P* = 0.738).

## Discussion

The present multicenter study retrospectively examined the screening efficiency of cytology alone, HR-HPV testing alone, and a combination of the two in a cohort of women with invasive cervical adenocarcinoma from China. We found that the sensitivity of cytology and HR-HPV co-testing was significantly higher than that of either test alone, and that there was no difference in the sensitivities of cytology alone and HR-HPV testing alone. These findings demonstrate that co-testing might be the most efficient method for invasive cervical adenocarcinoma screening.

Although the overall incidence and mortality rates of cervical cancer have been successfully reduced by cytological screening, the absolute incidence and proportion of cervical adenocarcinoma have risen in some developed countries in recent decades [3, 5]. A case–control study [32] of 188 paired specimens of adenocarcinoma and squamous cell carcinoma revealed that women with adenocarcinoma were more likely to have had false-negative cytology results than women with squamous cell carcinoma within the previous 2 years. A small number of retrospective studies [7, 24–26, 33] have shown that cytology alone is less efficient in identifying cervical adenocarcinoma than in identifying cervical squamous cell carcinoma. Moreover, a recent prospective controlled trial from Kaiser Permanente Northern California (KPNC) revealed that merely 14.8% (4/27) of adenocarcinomas were associated with an abnormal result on a prior conventional Papanicolaou smear test [15]. Although most patients (88.2%) in the present retrospective study were diagnosed within 1 month of the screening test (average, 37.8 days), the rate of positive results on cytology alone was only 64.0%. Thus, cytology had a high false-negative rate of 36.0% among patients with invasive adenocarcinoma. These results indicate that a screening strategy based solely on cytology might be insufficient to prevent invasive cervical adenocarcinoma.

There are several possible explanations for the ineffectiveness of cytological screening for cervical adenocarcinoma. First, cervical adenocarcinoma and its precancerous lesions are frequently located superior to the cervical transitional zone and mainly grow deep in the stroma of the endocervix. This may make it unlikely for the sampling spatula or brush to reach the malignant lesions and collect a sufficient number of exfoliated neoplastic cells for making a cancer diagnosis [33–35]. Second, glandular lesions are infrequently encountered in routine practice, leading to diagnostician unfamiliarity and interpretative challenges. Additionally, glandular neoplastic cells, especially in well-differentiated cervical adenocarcinomas, are likely to be mistaken for many benign conditions, such as metaplastic cells, reactive endocervical cells, endocervical cells with tubal metaplasia, and normal endometrial cells [33, 35, 36]. These situations might also lead to false-negative cytological interpretations. Moreover, as many as 25% of invasive cervical cancers, especially adenocarcinomas, can rapidly arise within an interval of less than 3 years between a normal cytology screening and the development of invasive cancer [27, 28]. These newly developed adenocarcinomas may not be detected in a timely manner during screening intervals.

HPV detection is widely or even independently used for primary cervical cancer screening because HR-HPV is closely related to cervical cancer [9, 10]. Many recent clinical studies [11–14] have confirmed that HR-HPV testing has a higher efficiency than cytology, albeit with lower specificity. However, several large-scale studies have shown that histologically confirmed cervical adenocarcinoma is less HPV-dependent than squamous cell carcinoma in paraffin-embedded samples [6, 19, 20, 22]. Moreover, adenocarcinoma represents a heterogeneous group with different histological subtypes, which are strongly, weakly, or irrelevantly related to HR-HPV infections [21–23, 37, 38]. Three large-scale studies [22, 23, 38] have reported similar results of HPV prevalence in different adenocarcinoma subtypes, with HPV-positive rates of more than 70% for the classic subtype and much lower detection rates for other morphological variants, such as gastric-type adenocarcinoma, serous carcinoma, and clear cell carcinoma.

Given the relatively low prevalence of HPV infection in cervical adenocarcinoma, we consider that the efficacy of HR-HPV detection for the screening of adenocarcinoma might be lower than that for screening squamous cell carcinoma. The aforementioned retrospective studies [7, 24–26] found that in adenocarcinoma screening, the HR-HPV-positive rates within 1 year before the primary cancer diagnosis ranged from 66.7 to 77.8%. A recent Chinese study by Jiang et al. [29] reported similar HR-HPV-positive rates (77.8%, 21/27) within 6 months before the adenocarcinoma diagnosis. Our present findings also showed a similar sensitivity of HR-HPV detection, with a prior HR-HPV-positive rate of 66% (95% CI: 57.0–75.1) for invasive cervical adenocarcinoma. The prospective KPNC study mentioned above showed that the baseline HR-HPV-positivity of adenocarcinomas was only 77.8% (21/27) [15]. Plausible reasons for the low screening efficacy of HR-HPV testing in the setting of invasive cervical adenocarcinoma are as follows: sampling variability and rapid progression, which are similar to the reasons for negative cytological screening; the test specimen may not contain a specific HPV genotype or may have a low viral load that cannot be detected by the detection assay [39, 40]; and the occurrence of truly HR-HPV-irrelevant cervical adenocarcinoma subtypes such as minimal deviation adenocarcinoma and clear cell carcinoma [22, 37, 38, 41].

Current data regarding which screening method—cytology or HPV detection—is more effective for cervical adenocarcinoma are limited and inconsistent. Our previous study [7] with a small number of cases showed that HR-HPV testing alone (77.8%) had similar sensitivity to cytology alone (73.7%) for adenocarcinoma screening. A study [26] from Quest Diagnostics in the United States has also reported similar results (cytology alone, 77.5% and HR-HPV testing alone, 73.4%). These results are consistent with those of our present multicenter study. The KPNC study [15] reported a much lower efficacy for cytology than for HR-HPV detection (14.8% vs. 77.8%). In contrast, two other results from China [24, 25] reported higher efficacy for cytology (94.4 and 77.8%) than for HPV testing (75.0 and 66.7%, respectively) in adenocarcinoma screening. However, the number of adenocarcinoma cases included in these two studies was small, and the statistical significance of their results was limited. Moreover, cervical screening is still not widely practiced in China, and at least some adenocarcinomas develop in patients without any prior screening. These cancers are larger and more advanced than the cancers seen in routine screening, which might make the cytological screening more sensitive.

Cytology and HR-HPV co-testing has been shown to not only significantly increase the efficiency of cervical cancer screening but also significantly reduce the overall incidence of invasive cancer and its precursors in subsequent screening rounds [15, 42]. Considering that HR-HPV testing may possibly be quite efficient in identifying HPV-positive cervical adenocarcinoma and that cytology may provide the means to detect HPV-negative adenocarcinoma variants and metastatic malignancies not driven by HPV infection, it is reasonable to believe that co-testing might be the most efficient screening method for adenocarcinoma. However, to date, no comprehensive data are available on co-testing for adenocarcinoma screening. In our previous study [7], co-testing showed obviously higher sensitivity in adenocarcinoma screening than cytology alone or HR-HPV testing alone; however, the difference was not statistically significant, mostly due to the small number of cases. Our present study further confirmed that co-testing had clearly higher sensitivity than cytology or HPV testing alone in adenocarcinoma screening. However, Katki et al. [15] reported that adding cytology to HPV testing did not further increase the efficiency of adenocarcinoma screening (77.8% vs. 77.8%). More large-scale screening results for adenocarcinoma should be obtained to verify if co-testing has the highest efficacy among these three screening strategies.

Although the data presented herein can offer further information for the selection of the optimal primary screening method for cervical adenocarcinoma, our study had several limitations. First, KingMed Diagnostics serves diverse hospitals and physical examination centers, mainly in suburbs and rural areas, where a large number of clinical providers receive no special training nor hold any special qualifications. The specifications for the sampling and preservation of the cervical specimens may not have been stringently applied, which may have led to inefficient cervical cancer screening. Nevertheless, our findings represent a large-scale experience in routine screening practices in China. Second, only patients with previous screening results within 6 months were included in this cohort, which may have led to selection bias. Third, most samples in this study were collected very close before the histological diagnosis of adenocarcinoma, and in some cases, the cytology and/or HPV tests were requested by clinicians because of a clinical suspicion of cervical adenocarcinoma. This bias may have made the cytology and/or HR-HPV testing appear to be more sensitive than it would have been during routine screening. Fourth, we had incomplete data on patient characteristics other than identity, age, time of cytology and/or HPV detection, and time of adenocarcinoma diagnosis. Therefore, we were unable to analyze the effects of different patient characteristics on the efficiency of the screening modalities. Fifth, the histological subtypes of cervical adenocarcinoma were not analyzed in the present study. Thus, whether there was a difference in the efficacy of cytology and/or HPV testing among different subtypes remains undetermined. Finally, a cost-effectiveness analysis of different screening tests was beyond the scope of this retrospective study. Further studies need to be done to determine which method is most appropriate for cervical adenocarcinoma screening in China.

## Conclusions

To the best of our knowledge, this study included the largest cohort of cervical adenocarcinoma patients with prior cervical cancer screening results from eight different pathology laboratories of KingMed Diagnostics from China. Thus, the study could provide a relatively robust analysis of the efficacy of cytology, HR-HPV testing, and the combination of cytology and HR-HPV testing in cervical adenocarcinoma screening in China. Our present data demonstrated that combined cytology and HR-HPV co-testing is a more sensitive and efficient strategy for the screening of invasive cervical adenocarcinoma than strategies based solely on cytology or HR-HPV testing. To maximize the detection of cervical adenocarcinoma, co-testing with cytology and HR-HPV detection should be recommended as the primary screening method in the forthcoming updated screening strategy for the prevention of cervical adenocarcinoma.

## Data Availability

All relevant data are within the paper. The data underlying this study are available and researchers may submit data requests to the the corresponding author on reasonable request.

## Abbreviations

AGC: Atypical glandular cells
ASC-H: Atypical squamous cells—cannot exclude HSIL
ASC-US: Atypical squamous cells of undetermined significance
HC2 assay: Hybrid Capture 2 assay
HR-HPV: High-risk human papillomavirus
HSIL: High-grade squamous intraepithelial lesion
KPNC: Kaiser Permanente Northern California
LSIL: Low-grade squamous intraepithelial lesion
NILM: Negative for intraepithelial lesion or malignancy
PCR: Polymerase chain reaction
